# Increased expression of FcγRI/CD64 on circulating monocytes parallels ongoing inflammation and nephritis in lupus

**DOI:** 10.1186/ar2590

**Published:** 2009-01-14

**Authors:** Yi Li, Pui Y Lee, Eric S Sobel, Sonali Narain, Minoru Satoh, Mark S Segal, Westley H Reeves, Hanno B Richards

**Affiliations:** 1 Division of Rheumatology & Clinical Immunology, University of Florida, 1600 SW Archer Road, Gainesville, FL 32610-0221, USA; 2 Division of Nephrology, Hypertension and Transplantation, Department of Medicine, University of Florida, 1600 SW Archer Road, Gainesville, FL 32610-0221, USA; 3 Schering-Plough Corporation, Kenilworth, NJ 07033-0530, USA

## Abstract

**Introduction:**

The high-affinity receptor for IgG Fcγ/CD64 is critical for the development of lupus nephritis (LN). Cross-linking Fc receptor on recruited monocytes by IgG-containing immune complexes is a key step in immune-complex-mediated nephritis in systemic lupus erythematosus (SLE). The goal of this study was to determine whether expression of Fc receptor (FcγR) I on circulating monocytes is associated with systemic inflammation and renal disease in SLE patients.

**Methods:**

We studied 205 SLE patients (132 with LN and 73 without LN) along with 74 healthy control individuals. Surface expression of CD14 (monocytes), FcγRI/CD64, FcγRII/CD32, and FcγRIII/CD16 was evaluated by flow cytometry. Monocyte function was assessed by determining the migratory capacity and the ability to produce CCL2 (monocyte chemotractic protein 1). High-sensitivity C-reactive protein, C3 and C4 were measured by nephelometry.

**Results:**

There was little difference in the expression of FcγRIII/CD16 or FcγRIII/CD32 on circulating monocytes between patients with SLE and control individuals. In contrast, FcγRI/CD64 expression was significantly higher in SLE patients and even higher in patients with LN. FcγRI/CD64 expression was positively associated with serum creatinine and indicators of systemic inflammation. Monocytes from patients with high FcγRI/CD64 expression also exhibited increased chemotaxis and capacity to produce monocyte chemotractic protein 1.

**Conclusions:**

Increased FcγRI/CD64 expression on circulating monocytes parallels systemic inflammation and renal disease in SLE patients. We propose that circulating monocytes activated by immune complexes and/or proinflammatory mediators upregulate surface expression of FcγRI/CD64 in SLE. The enhanced chemotactic and inflammatory potential of the activated monocytes may participate in a vicious cycle of immune cell recruitment and renal injury in SLE.

## Introduction

Systemic lupus erythematosus (SLE) is an autoimmune disease characterized by the production of autoantibodies against a wide array of self-antigens [1]. Formation of immune complexes (ICs) between these autoantibodies and the target antigens has been linked to the development of lupus nephritis (LN) [2,3]. Deposition of ICs in the kidneys activates monocyte/macrophages by interacting with Fc receptor (FcγR) I and FcγRIII, initiating an inflammatory cascade of cytokines and chemokines. The release of proinflammatory mediators such as monocyte chemotractic protein 1 (MCP-1 (CCL2)), macrophage inflammatory protein 1 (CCL3) and fractalkine (CX_3_CL1) recruits monocyte/macrophages and other immune effector cells, culminating in tissue damage [4,5].

Three classes of FcγRs are expressed on circulating human monocytes. FcγRI/CD64 is a high-affinity receptor constitutively expressed at substantial levels by monocytes [6]. Monocytes also express high levels of FcγRII/CD32, a low-affinity receptor for ICs with two functionally distinct isoforms. In contrast, FcγRIII/CD16, a receptor with moderate affinity for complexed IgG, is present on only about 10% to 15% of circulating monocytes [7]. FcγRI, FcγRIIa and FcγRIII are activating Fc receptors bearing intracytoplasmic tyrosine-based activation motifs that trigger monocyte activation upon receptor aggregation. FcγRIIb, on the other hand, contains an immunoreceptor tyrosine-based inhibitory motif and functions as an inhibitory Fc receptor upon interacting with ICs [8].

The balance of activating and inhibitory FcγR determines the magnitude of the cellular response in monocytes. Enhanced expression of activating FcγRs or decreased expression of the inhibitory FcγR can lower the activation threshold, leading to the production of inflammatory cytokines that may promote LN [9]. Conversely, NZB/W F1 mice deficient in FcγRI/III are protected from LN despite developing extensive IC deposits [10]. As in Wegener's granulomatosis [11] and rheumatoid arthritis [12], circulating monocytes in SLE are activated and exhibit increased surface expression of FcγRI/CD64 [13]. Whether this increase in activating FcγR on monocytes is related to development of LN, however, is unknown.

To investigate the possible role of activating FcγR in human LN, we examined the expression of FcγRI/CD64, FcγRIII/CD16 and FcγRII/CD32 on circulating monocytes from SLE patients, and the relationship of FcγR expression levels to renal involvement and chemokine production.

## Materials and methods

### Study population

The present study was approved by the University of Florida Institutional Review Board, and all subjects provided written informed consent prior to participation. SLE patients met at least four of the revised 1982 American College of Rheumatology criteria [14]. Peripheral blood was collected from 205 patients and 74 healthy control individuals. In the patient group, 132 participants had either biopsy-proven or laboratory-proven LN and 73 had no evidence of LN. At each visit a medication history and key laboratory parameters were collected. Disease activity was assessed using the Systemic Lupus Erythematosus Disease Activity Index [15]. Detailed demographics, clinical characteristics, medication usage and laboratory measurements for all groups are presented in Table 1.

**Table 1:** Demographics, laboratory characteristics and clinical characteristics of participants

	Control individuals (n = 74)	SLE patients without LN (n = 73)	SLE patients with LN (n = 132)
Demographics			
Female (%)	93	93	90
Mean age (years)	38	37	35
Race (%)			
African-American	37	31	43
Caucasian	32	49*	32^†^
Others	31	20	25
Disease duration (years)	-	9.0 ± 0.8	10.3 ± 0.8
American College of Rheumatology criteria count	-	6.0 ± 0.2	6.4 ± 0.2
Serum markers			
C3 (mg/dl)	125.1 ± 5.3	100.0 ± 3.7*	92.6 ± 5.0*
C4 (mg/dl)	24.7 ± 2.1	17.0 ± 1.1	19.4 ± 1.5
High-sensitivity C-reactive protein (mg/dl)	1.4 (1.1 to 4.4)	5.5 (4.1 to 7.0)*	5.8 (4.0 to 7.5)*
SLE manifestation^a ^(%)			
Central nervous system	-	21	14
Skin	-	65	53
Joint	-	87	68
Serositis	-	31	35
Anti-dsDNA	-	45	78^††^
Anti-Smith	-	40	57^†^
Anti-phospholipid	-	44	51
Medication usage^b ^(%)	-		
Prednisone	-	45	55
Mean dose (mg/day)	-	12.5	17.5
Antimalarials	-	80	72
Cytotoxics	-	28	68^††^
Statins	-	11	28^†^
Angiotensin-converting enzyme inhibitors	-	46	65^†^

^a^Presence of specific manifestations at any point during the course of disease. ^b^Medication usage at the time of this study. *P < 0.05 for systemic lupus erythematosus (SLE) patients with or without lupus nephritis (LN) versus healthy controls. ^†^*P *< 0.05 or ^††^*P *< 0.001 for SLE patients with LN versus SLE patients without LN.

### Cell surface staining

Antibodies were obtained from BD Pharmingen (San Diego, CA, USA) unless indicated otherwise. Heparinized whole blood (100 μl) was stained with PerCP-conjugated anti-CD14 (clone MΦ P9), fluorescein isothiocyanate-conjugated anti-CD16 (clone 3G8), allophycocyanin-conjugated anti-CD32 (clone FLI8.26), anti-HLA-DR (clone LN3), anti-CD62L (clone DREG56; eBioscience, San Diego, CA, USA), phycoerythrin-conjugated anti-CD64 (clone X54-5/7.1.1), and anti-CD16 (clone 3G8) for 30 minutes in the dark. Following lysis of erythrocytes, cells were washed with PBS/1% BSA/0.01% NaN_3 _and were fixed in 2% paraformaldehyde PBS. Cells (10^5^) were analyzed using a FACSCalibur flow cytometer and CellQuest software (Becton Dickinson, Mountain View, CA, USA).

Gates were set around monocytes based on their forward/sideward light scatter pattern and CD14 expression. Surface marker expression levels were expressed as the geometrical mean fluorescence intensity on monocytes. Since not all CD14 monocytes express CD16, CD32, CD62L and HLA-DR, expression was also expressed as the percentage of positive monocytes. Data analysis was performed using FCS Express 2.0 (De Novo Software, Thornhill, ON, Canada).

### Analysis of chemokine production

Heparinized whole blood was diluted 1:1 with DMEM (Mediatech, Inc., Herndon, VA, USA) containing 10% fetal bovine serum (Mediatech, Inc.), and was stimulated with lipopolysaccharide (LPS) (500 ng/ml, from *Escherichia coli*; Sigma Chemical Company, St Louis, MO, USA) or C-reactive protein (CRP) (50 ng/ml, purified from human serum, endotoxin-free; Calbiochem, La Jolla, CA, USA) in the presence of the protein transport inhibitor GolgiStop™ (BD Pharmingen). In all cases, cells were incubated at 37°C in a 5% CO_2 _atmosphere for 4 hours. The dose of LPS and CRP and the length of incubation were optimized for chemokine production in preliminary experiments. Immediately after incubation, 100 μl aliquots of cells were stained with appropriate combinations of monoclonal antibodies for 30 minutes at 22°C in the dark. After incubation, 2 ml PharMlyse (BD Pharmingen) was added to lyse erythrocytes. After washing, cells were fixed and permeabilized with 200 μl Cytofix/Cytoperm solution (BD Pharmingen) for 20 minutes at 4°C. After two washes with Perm/Wash solution (BD Pharmingen), cells were resuspended in 100 μl Perm/Wash solution containing 1.5 μg/μl phycoerythrin-conjugated anti-MCP-1 clone (5D3-F7; BD Pharmingen) or the same concentration of phycoerythrin-conjugated mouse IgG1 as an isotype control. After incubating at 4°C for 30 minutes in the dark, cells were washed and analyzed by flow cytometry.

### Chemotaxis assay

Peripheral blood mononuclear cells isolated from SLE patients and from healthy control individuals using Ficoll-Hypaque density gradient centrifugation were washed once and resuspended in DMEM containing 0.5% fetal bovine serum at a concentration of 10^7 ^cells/ml. Medium containing MCP-1 (25 ng/ml; Research Diagnostics Inc., Flanders, NJ, USA) or medium alone as a control were added to the lower chambers of a 24-well Costar Transwell plate (Corning Inc. Corning, NY, USA). The cell suspension (100 μl) was added to the upper chamber, which was separated from the lower chamber by a polycarbonate membrane (8.0 μm pores). After incubation for 3 hours at 37°C, cells in the lower chamber were collected, stained with anti-CD14, anti-CD16, and anti-HLA-DR, and analyzed by flow cytometry. Results are presented as a migration index calculated by dividing the number of cells that migrated toward MCP-1 by the number of cells that migrated to medium alone.

### Measurement of C-reactive protein and complement

High-sensitivity C-reactive protein, C3 and C4 assays were performed using a BN ProSpec^® ^Nephelometer (Dade Behring, Deerfield, IL, USA) as described elsewhere [16].

### *In vitro *stimulation of healthy donor peripheral blood mononuclear cells

Peripheral blood mononuclear cells from healthy control individuals were plated on 24-well plates (10^6 ^cells/well) in complete medium (DMEM supplemented with 10% fetal bovine serum, 20 mM L-glutamine, 100 IU/ml penicillin, and 100 μg/ml streptomycin). All cytokines were from BD Bioscience unless indicated otherwise. Cells were incubated for 19 hours at 37°C in the presence of recombinant human IFNα (4 ng/ml; PBL Biomedical, Piscataway, NJ, USA), IFNγ (2 ng/ml), IL-4 (4 ng/ml), IL-6 (4 ng/ml), IL-8 (4 ng/ml), IL-12 (4 ng/ml), or CRP (50 ng/ml; Calbiochem). Flow cytometry was performed immediately after incubation. In some experiments, dexamethasone (10^-5 ^to 10^-3 ^M) was added to the culture 3 hours prior to the addition of cytokines.

### Statistical analysis

Differences between disease groups and normal control individuals were evaluated using Student's two-tailed *t *test unless the data were not normally distributed, in which case the Mann–Whitney *U *test was used. Correlation coefficients were calculated using Spearman's rank correlation. Data are presented as the mean ± standard error of the mean. Analyses were performed using Prism software, version 4.0 (GraphPad Software, San Diego, CA, USA). For all analyses, *P *< 0.05 was considered significant.

## Results

We assessed the surface expression of FcγRs on monocytes from SLE patients with or without LN and from healthy control individuals. Demographics and clinical/laboratory data are summarized in Table 1. There was no difference in the percentage of circulating CD14^+ ^monocytes between SLE patients with or without LN and normal control individuals (Table 2). Absolute monocyte counts, however, were significantly decreased in SLE patients with/without LN when compared with normal control individuals (268 ± 29 cells/μl and 254 ± 39 cells/μl, respectively, versus 357 ± 32 cells/μl; both *P *< 0.005, Student's *t *test).

**Table 2:** Comparison of cell surface marker expression by CD14^+ ^monocytes

	Control individuals	SLE patients without LN	SLE patients with LN
CD14^+ ^cells			
Percentage^a^	4.3 ± 0.4	3.9 ± 0.3	4.0 ± 0.2
Mean fluorescence intensity	596.8 ± 42.9	604.8 ± 29.9	605.3 ± 18.7
Absolute number (cell/μl)	357.2 ± 31.5	254.1 ± 38.5*	268.3 ± 29.4*
FcγRIII/CD16			
Percentage^a^	9.3 ± 0.7	11.0 ± 0.6*	10.8 ± 0.6
Mean fluorescence intensity	10.2 ± 0.6	13.2 ± 0.6**	12.5 ± 0.4*
Absolute number (cell/μl)	23.3 ± 4.1	53.6 ± 10.0	78.9 ± 15.7
FcγRII/CD32			
Percentage^a^	87.5 ± 2.9	85.9 ± 2.3	81.8 ± 2.9
Mean fluorescence intensity	71.6 ± 8.3	64.4 ± 7.5	68.4 ± 5.9
Absolute number (cell/μl)	266.7 ± 49.2	249.1 ± 114.5	238.2 ± 100.9
FcγRI/CD64			
Percentage^a^	99.9 ± 0.03	99.9 ± 0.02	99.9 ± 0.03
Mean fluorescence intensity	319.1 ± 22.2	449.2 ± 30.5**	567.0 ± 28.1**^††^
Absolute number (cell/μl)	352.8 ± 0.11	252.8 ± 0.05*	268.0 ± 0.08*

*P < 0.05 or **P < 0.001 for systemic lupus erythematosus (SLE) patients with or without lupus nephritis (LN) versus healthy control individuals. ^†^*P *< 0.05 or ^††^*P *< 0.001 for SLE patients with LN versus SLE patients without LN.

### Increased FcγRI/CD64 expression on SLE monocytes

**Figure 1:**
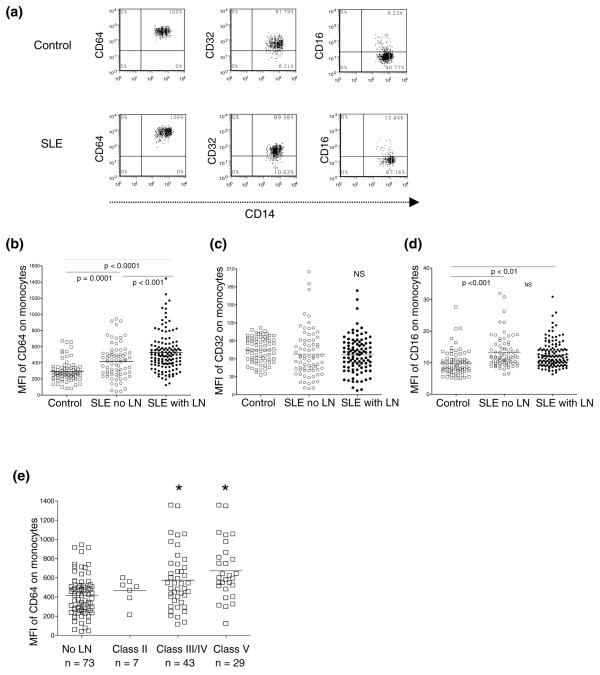
**Expression of Fc receptors in healthy control individuals versus systemic lupus erythematosus patients**. **(a) **Representative scattergrams of surface expression of FcγRI/CD64, FcγRII/CD32 and FcγRIII/CD16 on CD14^+ ^monocytes from a healthy control individual (top) and a patient with systemic lupus erythematosus (SLE) (bottom). CD14^+ ^monocytes were gated based on their forward/sideward scatter. **(b-d) **Expression of FcγRI/CD64, FcγRII/CD32 and FcγRIII/CD16, respectively (mean fluorescence intensity (MFI)) on SLE versus control monocytes. SLE patients with and without lupus nephritis (LN) are analyzed separately. Differences between groups were compared by Student's *t *test. **(e) **Comparison of FcγRI/CD64 expression (MFI) on monocytes from SLE patients without LN or with biopsy-proven World Health Organization class II, class III/IV, or class V LN. *P < 0.05 compared with SLE patients without LN (Student's *t *test).

In healthy control individuals, nearly all peripheral blood monocytes displayed surface expression of FcγRI/CD64 and FcγRII/CD32. Only 9.3 ± 0.7% of circulating monocytes, however, expressed FcγRIII/CD16 (Figure 1a, top and Table 2). Although circulating monocytes from SLE patients also uniformly expressed FcγRI/CD64 (Figure 1a, bottom), quantification of FcγRI/CD64 expression showed a significantly higher mean fluorescence intensity in SLE patients compared with healthy control individuals (521 ± 21 versus 319 ± 22; *P *< 0.001, Student's *t *test). The expression was even higher in patients with nephritis compared with those without nephritis (567 ± 28 versus 449 ± 31; *P *< 0.001, Student's *t *test) (Figure 1b and Table 2).

In contrast, CD32 expression was similar on CD14^+ ^monocytes from SLE patients versus normal healthy control individuals (Figure 1c and Table 2). While the frequencies and absolute numbers of CD16^+^CD14^+ ^monocytes were similar between SLE patients and control individuals, the intensity of CD16 staining was increased slightly in SLE patients with or without LN (12 ± 0.4 and 13 ± 0.6, respectively, versus control individuals 10 ± 0.6; both *P *< 0.01, Student's *t *test) (Figure 1d and Table 2). We also assessed the expression of HLA-DR and CD62L, markers related to monocyte activation, but found no significant differences between the groups (data not shown).

To further evaluate the relationship between FcγR expression and LN, we analyzed the expression of FcγRs on monocytes in 79 patients who had undergone renal biopsy (class II, n = 7; class III/IV, n = 43; and class V, n = 29). The presence of class III/IV or class V LN, but not of class II LN, was associated with increased expression of FcγRI/CD64 compared with SLE patients who did not have LN (Figure 1e). In contrast, the expression of FcγRII and FcγRIII was similar among the different classes of LN (data not shown).

### Increased FcγRI/CD64 expression is associated with impaired renal function

**Figure 2:**
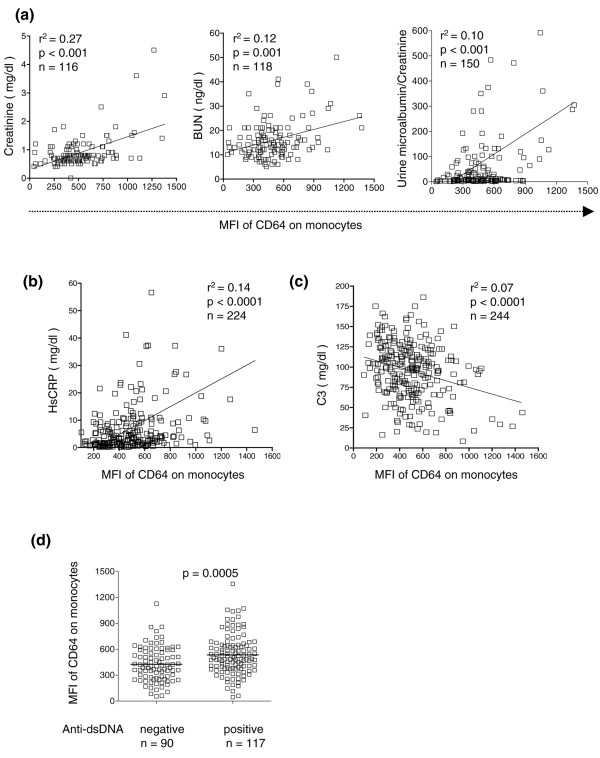
**FcγRI/CD64 expression on monocytes correlates with renal disease, C-reactive protein, and complement C3 levels**. **(a) **FcγRI/CD64 expression levels on circulating monocytes from systemic lupus erythematosus (SLE) patients (both lupus nephritis (LN) and non-LN patients) correlated with increased serum creatinine (left) and blood urea nitrogen (BUN) (middle), as well as with proteinuria (microalbumin/creatinine ratio) (right). MFI, mean fluorescence intensity. Expression of FcγRI/CD64 correlated **(b) **positively with serum high-sensitivity C-reactive protein (HsCRP) levels and **(c) **negatively with serum C3 levels (Spearman's correlation). **(d) **Comparison of FcγRI/CD64 expression in SLE patients positive or negative for anti-dsDNA autoantibodies (Student's *t *test).

Since FcγRI/CD64 expression on monocytes was greater in SLE patients with LN compared with SLE patients without LN, we investigated its relationship with individual markers of renal involvement. Increased expression of FcγRI/CD64 on monocytes correlated positively with elevated creatinine (*r*^2 ^= 0.27, *P *< 0.001; Spearman's correlation) (Figure 2a, left) and blood urea nitrogen levels (*r*^2 ^= 0.12, *P *= 0.001; Spearman's correlation) (Figure 2a, middle), as well as with the degree of proteinuria (microalbumin/creatinine ratio, *r*^2 ^= 0.10, *P *< 0.001; Spearman's correlation) (Figure 2a, right).

### Increased levels of FcγRI/CD64 expression are associated with ongoing inflammation

We next examined the relationship of FcγRI/CD64 expression with measures of systemic inflammation such as high-sensitivity CRP and complement C3 [17]. In patients with SLE, the expression of FcγRI/CD64 on monocytes was positively correlated with elevated serum levels of high-sensitivity CRP (*r*^2 ^= 0.14, *P *< 0.0001; Spearman's correlation) (Figure 2b). FcγRI/CD64 expression showed an inverse relationship with serum C3 (*r*^2 ^= 0.07, *P *< 0.0001; Spearman's correlation) (Figure 2c) but not with C4 (*r*^2 ^= 0.01, *P *= 0.14) (data not shown). Increased FcγRI/CD64 expression was also associated with anti-dsDNA autoantibodies (534.1 ± 21.4 versus 426.5 ± 21.0 mean fluorescence intensity units, *P *= 0.0005) (Figure 2d). Increased FcγRI/CD64 surface expression on monocytes was therefore associated with impaired renal function, anti-dsDNA autoantibody production, C3 consumption, and ongoing inflammation in SLE patients.

### FcγRI/CD6^hi ^monocytes have an activated phenotype

**Figure 3:**
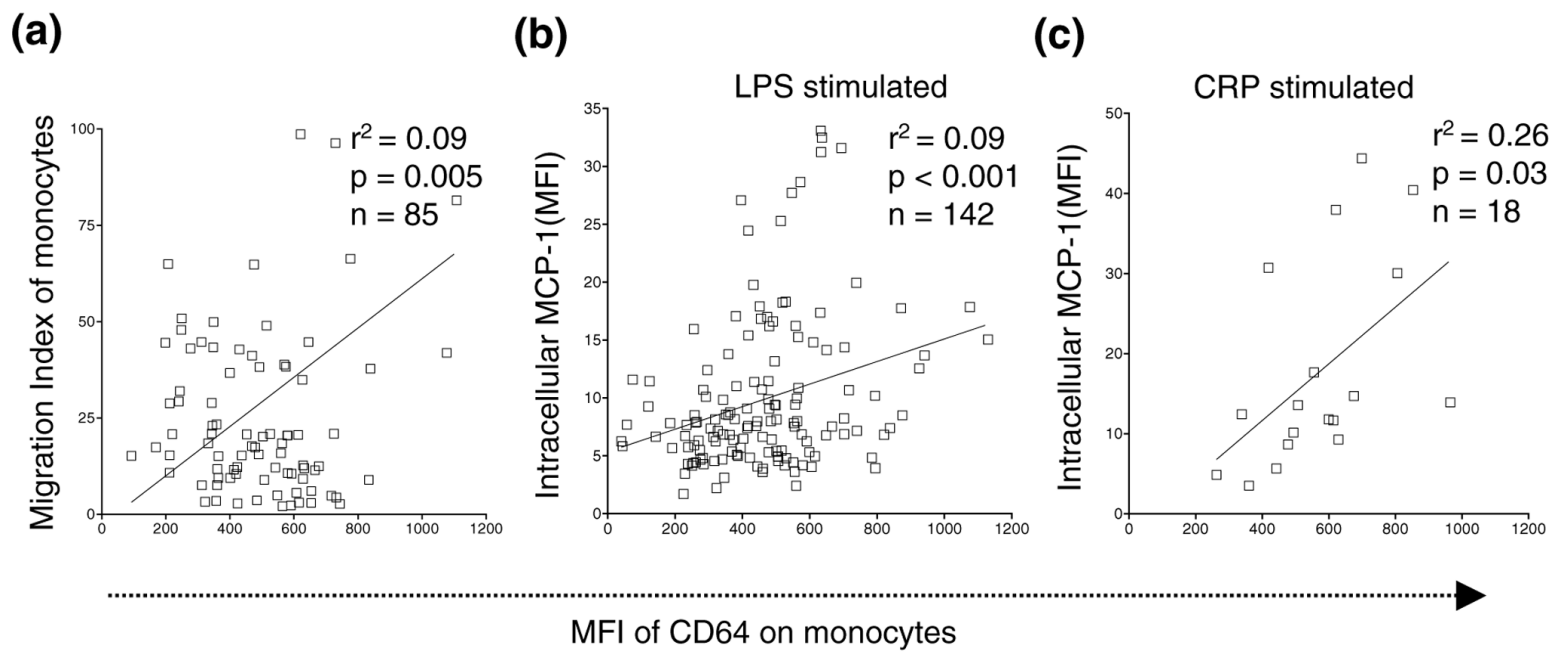
**FcγRI/CD64^hi ^monocytes have an activated phenotype**. **(a) **Correlation between FcγRI/CD64 expression levels and monocyte migration toward monocyte chemotractic protein 1 (MCP-1) (transwell assay, systemic lupus erythematosus (SLE) patients). **(b) **Correlation of elevated FcγRI/CD64 expression on monocytes with an increased capacity to produce MCP-1, as measured by intracellular staining of CD14^+ ^monocytes 4 hours after lipopolysaccharide (LPS) stimulation. MFI, mean fluorescence intensity. **(c) **Correlation of FcγRI/CD64 expression with levels of MCP-1 production by monocytes following C-reactive protein (CRP) stimulation for 4 hours.

Monocyte migration to the kidneys and the subsequent release of inflammatory mediators are thought to be critical steps initiating renal damage [18,19]. We evaluated the migratory capacity of circulating monocytes from SLE patients using an *in vitro *transwell assay, and found that monocytes with elevated FcγRI/CD64 expression exhibited increased migration toward the chemokine MCP-1 (*r*^2 ^= 0.09, *P *= 0.005; Spearman's correlation) (Figure 3a).

As monocyte-derived proinflammatory cytokines and chemokines such as MCP-1 regulate immune cell infiltration and play an important role in organ damage in SLE [20], we examined the ability of CD64^+ ^monocytes to produce MCP-1. After LPS stimulation, monocytes with high FcγRI/CD64 expression produced higher levels of the chemokine than CD64^- ^monocytes, as measured by intracellular staining (*r*^2 ^= 0.09, *P *< 0.001; Spearman's correlation) (Figure 3b).

Since the binding of CRP to FcγRI/CD64 and FcγRIIa/CD32a can lead to increased inflammatory cytokine production [21-23], we stimulated monocytes from SLE patients with CRP (50 ng/ml) and analyzed the MCP-1 production. CRP and LPS elicited similar levels of intracellular MCP-1 staining (compare Figure 3b and Figure 3c). Consistent with the results with LPS stimulation, high FcγRI/CD64 surface expression was associated with increased intracellular MCP-1 production in response to CRP (*r*^2 ^= 0.26, *P *= 0.03; Spearman's correlation) (Figure 3c). Monocytes with elevated surface expression of FcγRI/CD64 therefore displayed a more activated phenotype in terms of migratory properties and MCP-1 production in response to either LPS or CRP.

### Medication effects on FcγRI/CD64 expression

**Figure 4:**
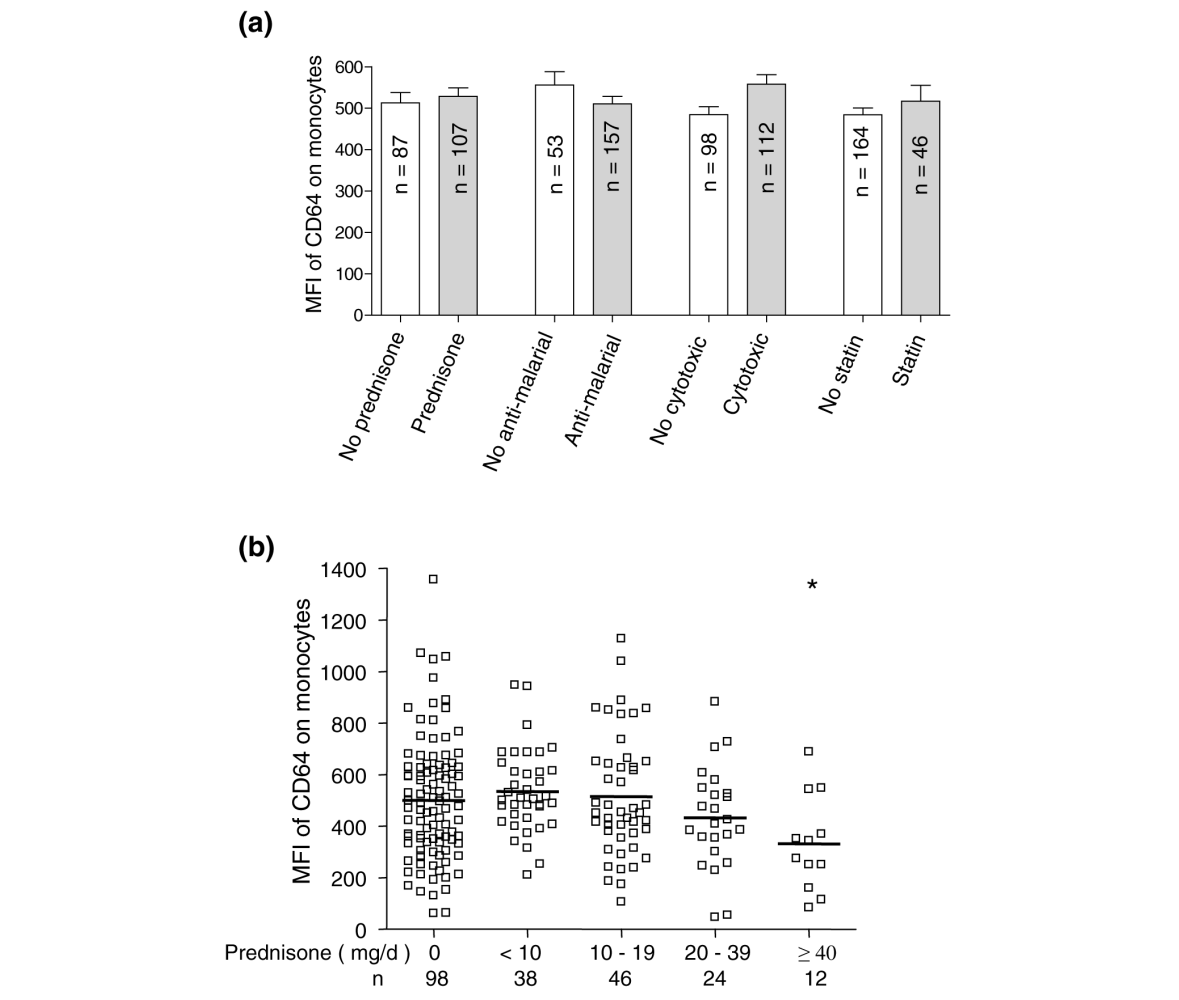
**Effect of medications and cytokines on FcγRI/CD64 expression by circulating monocytes**. **(a) **Comparison of FcγRI/CD64 expression on monocytes between systemic lupus erythematosus (SLE) patients receiving or not receiving prednisone, antimalarials, cytotoxic drugs, or statins. MFI, mean fluorescence intensity. **(b) **Relationship between daily corticosteroid dose and monocyte FcγRI/CD64 expression in SLE patients. *P < 0.05 compared with SLE patients not receiving steroid treatment.

Corticosteroids potently downmodulate certain inflammatory markers on circulating monocytes [24]. Since about one-half of our SLE patients were treated with corticosteroids (Table 1), we asked whether the levels of FcγRI/CD64 expression by monocytes were affected by treatment. When analyzed as a group, patients treated with conventional doses of prednisone (< 40 mg/day) showed no difference in FcγRI/CD64 expression compared with those patients not treated with corticosteroids (Figure 4a). There also was no apparent effect of antimalarial, cytotoxic or statin therapy on the expression of FcγRs (Figure 4a). Stratifying patients based on the prednisone dose revealed that a daily dosage ≥ 40 mg was associated with decreased FcγRI/CD64 expression on monocytes (Figure 4b). A similar trend (not statistically significant) was seen at a dose of 20 to 30 mg/day. This effect was not seen at lower dosages (Figure 4b). Patients treated with ≥ 40 mg/day prednisone tended to display lower serum levels of C3 (67.6 ± 8.5 versus 93.7 ± 3.4 mg/dl; *P *< 0.05) and higher levels of blood urea nitrogen (36.9 ± 9.0 versus 15.6 ± 0.8 ng/dl; *P *< 0.05) compared with their counterparts given lower doses, consistent with higher disease activity (data not shown). There was no difference in Systemic Lupus Erythematosus Disease Activity Index scores, American College of Rheumatology criteria counts, serum creatinine, high-sensitivity CRP levels, or microalbumin/creatinine ratios between the groups (data not shown).

### Effect of cytokines on FcγRI/CD64 expression

**Figure 5:**
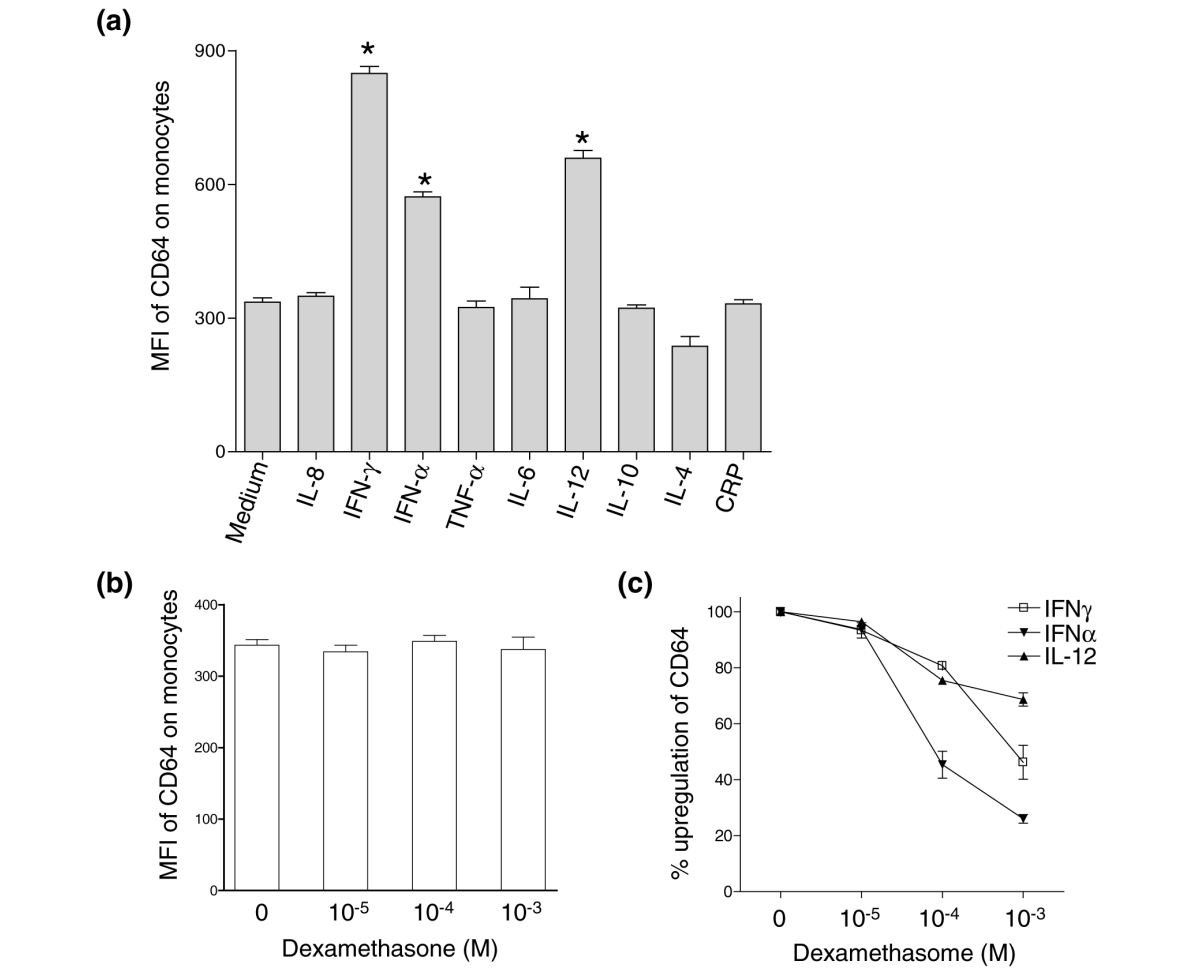
**Effect of cytokines and dexamethasone on FcγRI/CD64 expression *in vitro***. **(a) **Direct effects of cytokines and C-reactive protein (CRP) on FcγRI/CD64 expression on circulating monocytes. Peripheral blood mononuclear cells from healthy subjects were cultured with recombinant IFNα (4 ng/ml), IFNγ (2 ng/ml), IL-4 (4 ng/ml), IL-6 (4 ng/ml), IL-8 (4 ng/ml), IL-12 (4 ng/ml) or CRP (50 ng/ml) for 19 hours *in vitro*. FcγRI/CD64 expression (mean fluorescence intensity (MFI)) was analyzed by flow cytometry. Values represent the mean ± standard error of the mean (SEM) from five independent experiments. *P < 0.001 compared with medium alone. **(b) **Effect of dexamethasone on monocyte FcγRI/CD64 expression *in vitro*. Values represent the mean ± SEM from three independent experiments. **(c) **Effect of dexamethasone on the upregulation of FcγRI/CD64 by IFNα, IFNγ, and IL-12. Values represent the mean ± SEM from two independent experiments.

Several studies have shown that the expression of FcγRI/CD64 can be influenced by different cytokines in pathogenic circumstances. Dysregulation of proinflammatory cytokine production has also been well documented in SLE. To examine potential inducers of FcγRI/CD64 upregulation, we stimulated peripheral blood mononuclear cells from healthy control individuals with a panel of cytokines. Overnight incubation with IFNα, IFNγ, and IL-12 significantly increased FcγRI/CD64 expression on monocytes, whereas IL-6, IL-8, IL-10, TNFα, and CRP treatment did not (Figure 5a). Similar results were obtained when the experiment was performed using cultured THP-1 cells (data not shown).

Curiously, while the addition of dexamethasone to whole blood did not alter the steady-state levels of FcγRI/CD64 expression on monocytes *in vitro *(Figure 5b), high concentrations of dexamethasone (≥ 10^-4 ^M) inhibited the upregulation of FcγR1/CD64 expression induced by IFNγ, IFNα and IL-12 (Figure 5c). This effect was not seen with lower concentrations of dexamethasone.

## Discussion

In mouse models of SLE, monocytes/macrophages bearing activating Fc receptors are pivotal to the development of IC-mediated glomerulonephritis [25,26]. There is indirect evidence that the same may be true of human lupus [27,28], although the relationship between activating FcγR expression and the pathogenesis of human LN is less clear than in the mouse. In the present study, we examined FcγR expression in more than 200 SLE patients. The levels of FcγRI/CD64 expression on circulating monocytes were significantly elevated in SLE patients, especially in those with LN. Increased monocyte FcγRI/CD64 expression also was associated with markers of impaired renal function impairment and with a greater ability to migrate and secrete the chemokine MCP-1.

The proinflammatory role of activating FcγR in LN is evident in mice deficient in FcγRI/III, which are protected from the development of renal disease despite the presence of glomerular IC deposits [10]. A recent study showed that the expression of FcγRI/III by monocytes was both necessary and sufficient to trigger nephritis in NZB/W F1 mice [26]. In contrast, the inhibitory FcγRIIb suppresses inflammation and spontaneous activation of autoreactive lymphocytes and autoantibody production in mice [26,29].

In human SLE, several groups have shown the abnormal upregulation of activating Fcγ receptors on monocytes [13,30]. One relatively small study, however, found no significant difference in FcγRI/CD64 or FcγRIII/CD16 expression on SLE monocytes compared with healthy controls [31]. About two-thirds of the patients studied here had elevated levels of monocyte surface CD64 in the present study, a discrepancy that may be due to the relatively small number of subjects studied previously. Consistent with the observations of others [13,28], our data show that the activating receptor FcγRIII/CD16 also is upregulated in SLE patients compared with healthy control individuals. In line with murine lupus data, our data support the idea that activating FcγRs play a crucial role in IC-mediated organ damage in SLE.

Although NZB/W F1 mice deficient in activating FcγRs are protected from renal disease, the relative contributions of the individual activating FcγRs have not been studied further. Our data show that although both FcγRIII/CD16 and FcγRI/CD64 expression were elevated, increased FcγRIII/CD16 expression was not associated with LN, suggesting that activation via FcγRI/CD64 may be more significant to the pathogenesis of human LN. Moreover, we found no difference in the surface expression of FcγRII/CD32 on monocytes between the SLE patients and healthy control individuals, although interpretation of this finding is limited by the inability of the anti-CD32 antibody to distinguish the activating FcγRIIa and inhibitory FcγRIIb. Expression of the inhibitory FcγRIIb in peripheral blood mononuclear cells from SLE patients has been recently studied using specific antibodies [32]. While low expression levels were found on B-lymphocyte subsets, FcγRIIb/CD32b expression was not impaired on monocytes from SLE patients.

The importance of FcγRs in the pathogenesis of SLE is further illustrated by extensive polymorphism studies involving FcγRII/CD32 and FcγRIII/CD16. Several of these polymorphisms – including FcγRIIa-131R, FcγRIIIa-176F, and FcγRIIIb-NA2 – have been associated with lupus susceptibility [33,34]. Importantly, some of them cause functional alterations of the inhibitory receptor [35,36] while others are associated with reduced surface expression of FcγRIIb on both memory and plasma B lymphocytes [37]. To our knowledge, however, polymorphisms involving FcγRI/CD64 have not been linked to SLE.

The markedly elevated expression of FcγRI/CD64 among SLE patients with LN (Figure 1b) may serve as a surrogate marker of renal disease that correlates with both established measures of renal dysfunction (increased serum creatinine, blood urea nitrogen, and proteinuria) and inflammation (elevated serum CRP, C3 deficiency). Monocyte FcγRI/CD64 expression, however, did not correlate with overall disease activity as assessed by the Systemic Lupus Erythematosus Disease Activity Index (data not shown). This was not due to medication use, since FcγRI/CD64 levels on circulating monocytes were unaffected by treatment with prednisone at doses < 40 mg/day, or by antimalarials, cytotoxic agents, or statins. In contrast, higher doses of prednisone (≥ 40 mg/day) or dexamethasone treatment *in vitro *reduced FcγRI/CD64 expression, possibly due to direct effects on proinflammatory cytokine production [38,39] or to the generation of a subset of anti-inflammatory monocytes that secrete IL-10 [40,41].

Our *in vitro *data suggest that IL-12, IFNγ, and IFNα are potential inducers of FcγRI/CD64 expression in SLE. Interestingly, excess production of all three of these cytokines promotes LN in mice [42-44]. In human LN, increased levels of IFNγ, IL-12 and IFNα/β are found in the kidney [45,46]. Dysregulation of IFNα production is also associated with renal involvement [47]. Our data are consistent with the possibility that the overproduction of one or more of these cytokines promotes LN by enhancing the recruitment of proinflammatory (CD64^+^) monocytes/macrophages to the renal glomerulus. Although there was a highly significant correlation between FcγRI/CD64 expression and several markers of renal involvement or inflammation (Figure 2), the *r*^2 ^values were in some cases relatively low. This indicated the existence of additional variables, at present undefined, affecting FcγRI/CD64 expression. Elucidating the variables that affect FcγRI/CD64 expression, perhaps including serum levels of the cytokines examined in our in *vitro *studies, will require further study.

FcγRI/CD64 plays a role in phagocytosis, cytolysis, degranulation, and induction of inflammatory cytokines. Additionally, FcγRI-deficient mice display defective peritoneal monocyte infiltration in response to ICs [48]. Consistent with these studies, our data demonstrated that circulating human monocytes from patients with upregulated FcγRI/CD64 expression exhibited increased migratory capacity and MCP-1 production in response to LPS or CRP stimulation. Monocyte/macrophage infiltration is important in promoting mesangial hypercellularity and the development of glomerulosclerosis in both human and animal models [49,50]. Additionally, the number of infiltrating monocytes/macrophages is associated with more severe renal injury and poor prognosis in LN [50,51].

As seen in animal models [10,26,52], monocytes expressing FcγRI/CD64 may be important to the pathogenesis of IC-mediated nephritis in SLE. Elevated production of IFNα and IFNγ in SLE may induce the expression of FcγRI/CD64 monocytes and facilitate the infiltration of these cells to the sites of IC deposition in the kidney [48]. Since IFNα and IFNγ also stimulate the production of monocyte attractants such as MCP-1, the presence of these cytokines in the kidney also may promote the influx of monocytes. In turn, signal transduction downstream of FcγRI/CD64 leads to monocyte activation and further production of inflammatory cytokines and chemokines. These events could culminate in a vicious cycle of renal inflammation and monocyte infiltration, ultimately leading to permanent tissue damage.

## Conclusion

Our study demonstrates that elevated surface expression of FcγRI/CD64 is associated with ongoing systemic inflammation and renal disease in lupus patients. We propose that upregulation of FcγRI/CD64 expression on circulating monocytes may be a useful surrogate marker of monocyte activation in SLE.

## Abbreviations

BSA: bovine serum albumin; CRP: C-reactive protein; DMEM: Dulbecco's modified Eagle's medium; FcγR: Fcγ receptor; IC: immune complex; IFN: interferon; IL: interleukin; LN: lupus nephritis; LPS: lipopolysaccharide; MCP-1: monocyte chemotractic protein 1; PBS: phosphate-buffered saline; SLE: systemic lupus erythematosus; TNF: tumor necrosis factor.

## Competing interests

The authors declare that they have no competing interests.

## Authors' contributions

WHR and HBR contributed equally to this work. YL carried out data analysis and interpretation, and the study design, and assisted in manuscript preparation. PYL participated in date analysis and interpretation, and assisted in manuscript preparation. ESS participated in acquisition of data and patient recruitment. SN participated in statistical analysis. MS assisted in data interpretation. MSS participated in acquisition of data and patient recruitment. WHR carried out the study design and data interpretation, and assisted in patient recruitment and preparation of the manuscript. HBR conceived of the study and coordinated patient recruitment, data analysis and preparation of the manuscript. All authors read and approved the final manuscript.
